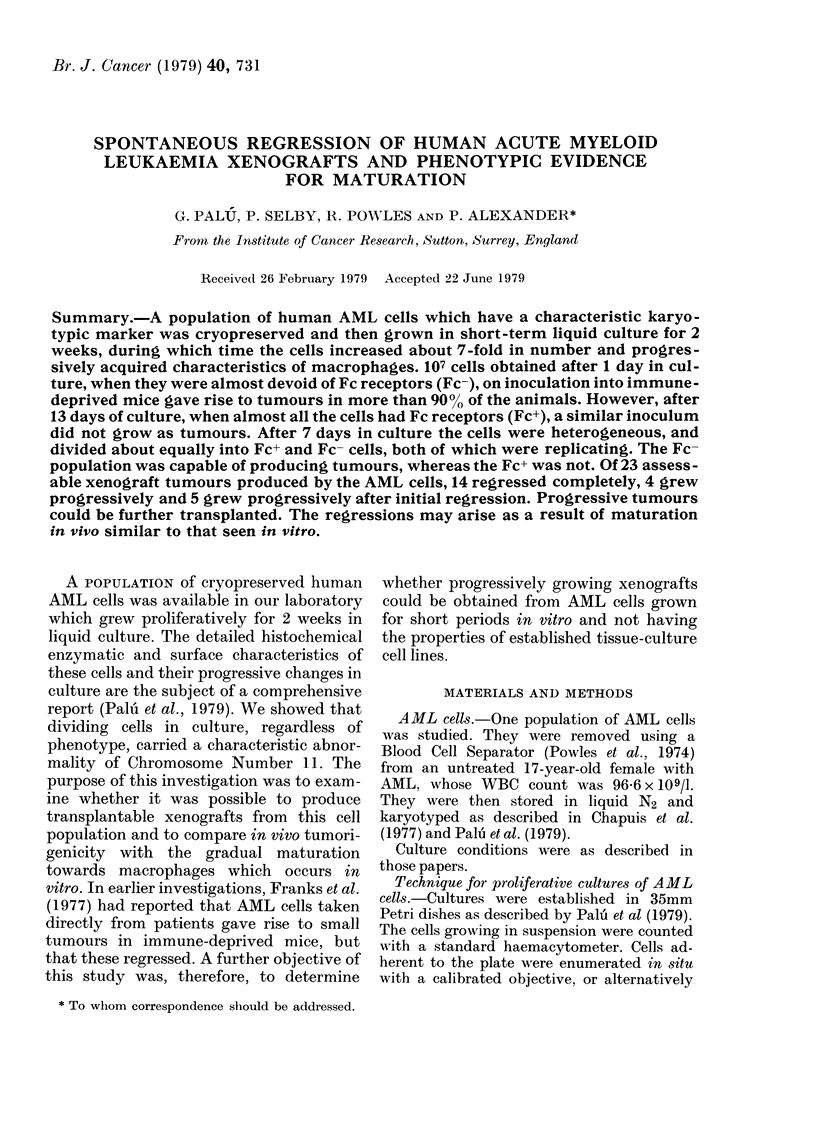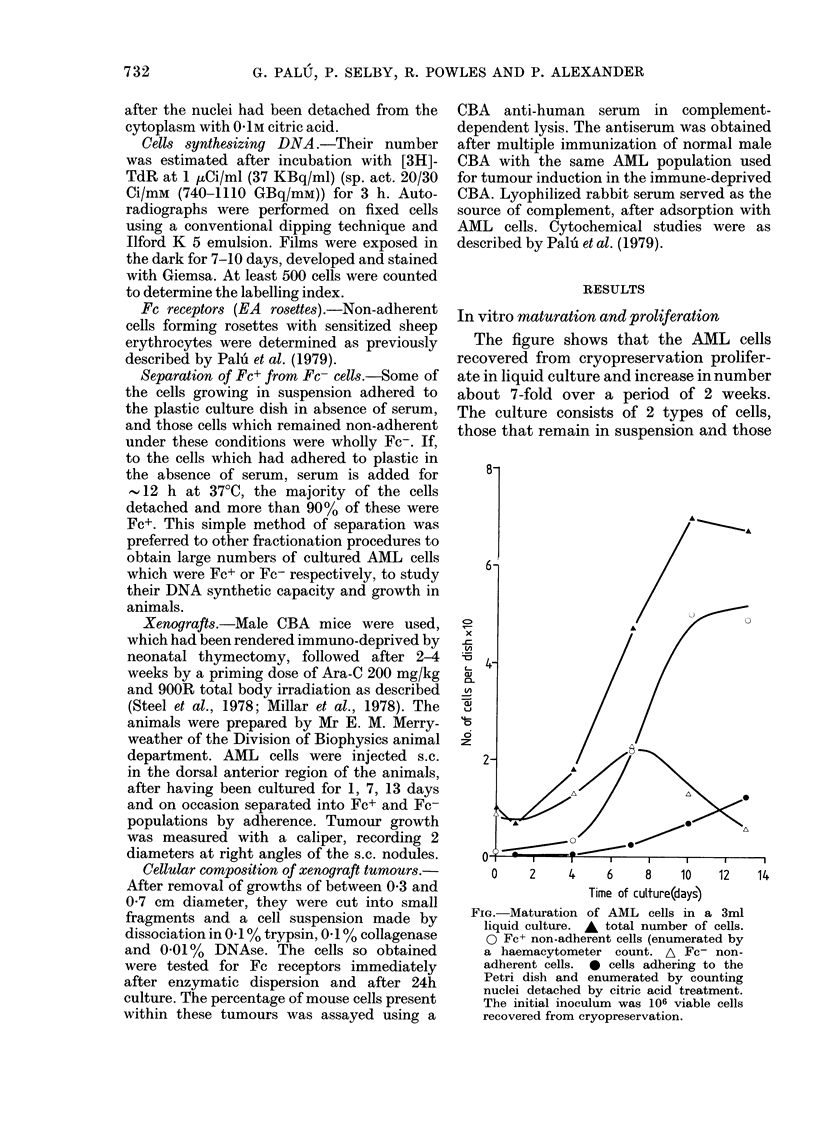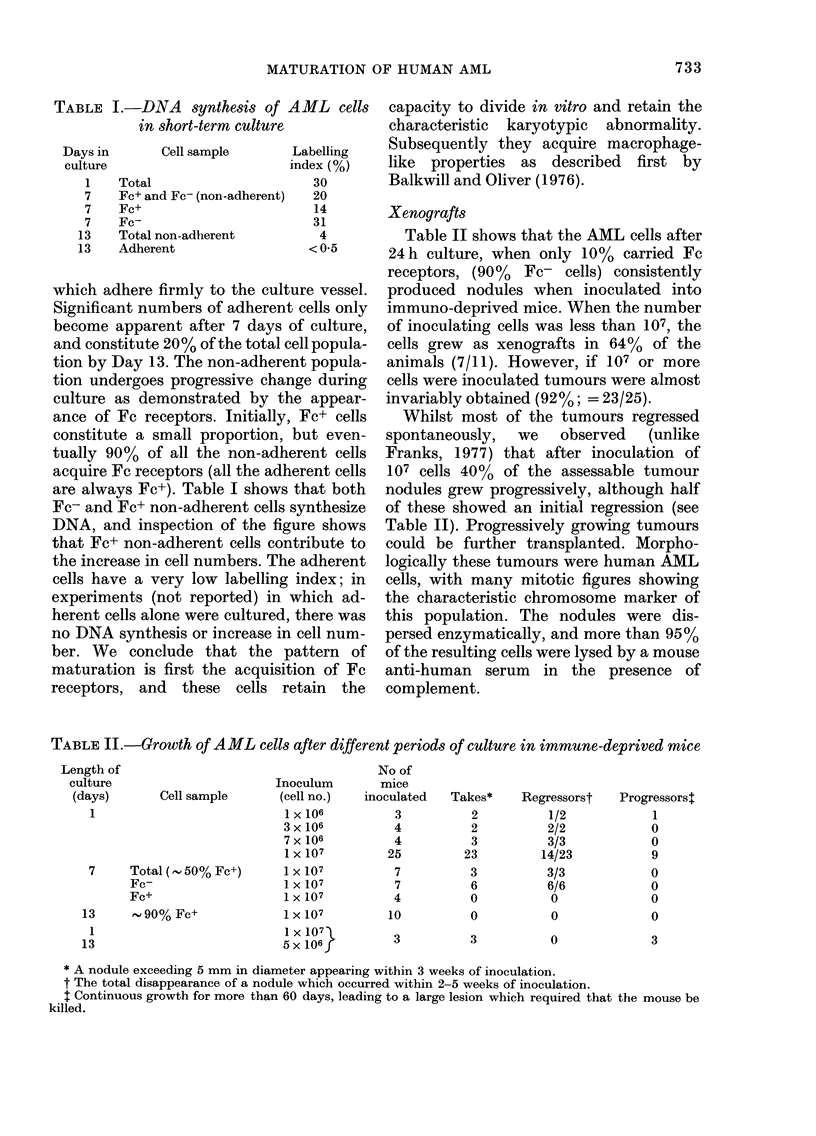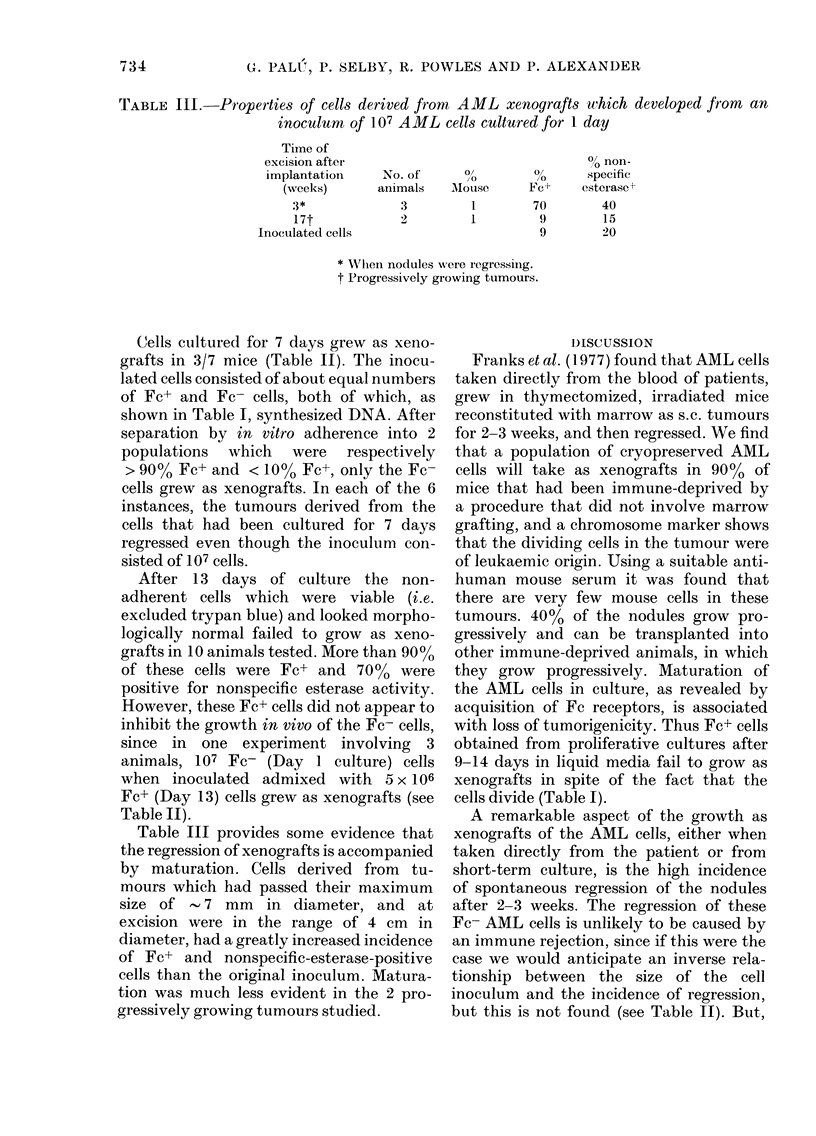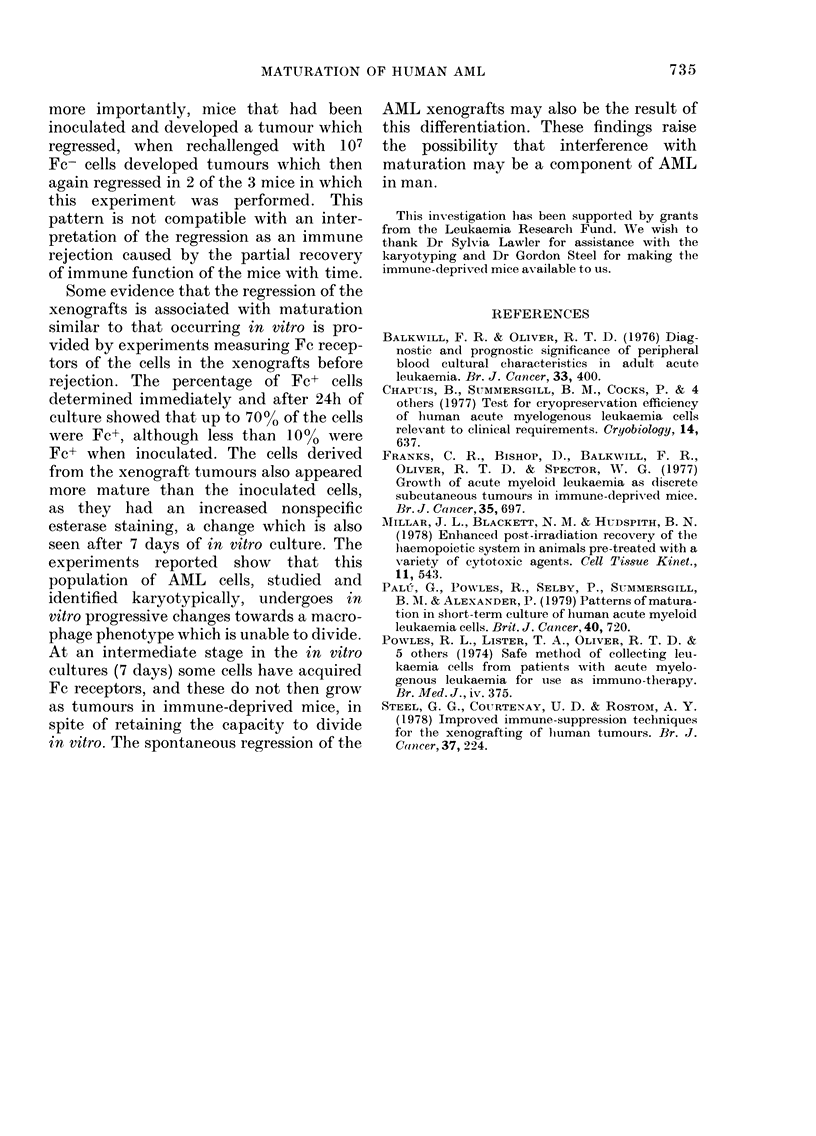# Spontaneous regression of human acute myeloid leukaemia xenografts and phenotypic evidence for maturation.

**DOI:** 10.1038/bjc.1979.253

**Published:** 1979-11

**Authors:** G. Palú, P. Selby, R. Powles, P. Alexander

## Abstract

A population of human AML cells which have a characteristic karyotypic marker was cryopreserved and then grown in short-term liquid culture for 2 weeks, during which time the cells increased about 7-fold in number and progressively acquired characteristics of macrophages. 10(7) cells obtained after 1 day in culture, when they were almost devoid of Fc receptors (Fc-), on inoculation into immune-deprived mice gave rise to tumours in more than 90% of the animals. However, after 13 days of culture, when almost all the cells had Fc receptors (Fc+), a similar inoculum did not grow as tumours. After 7 days in culture the cells were heterogeneous, and divided about equally into Fc+ and Fc- cells, both of which were replicating. The Fc- population was capable of producing tumours, whereas the Fc+ was not. Of 23 assessable xenograft tumours produced by the AML cells, 14 regressed completely, 4 grew progressively and 5 grew progressively after initial regression. Progressive tumours could be further transplanted. The regression may arise as a result of maturation in vivo similar to that seen in vitro.


*Br. J. Cancer* (1979) **40**, 731

### SPONTANEOUS REGRESSION OF HUMAN ACUTE MYELOID LEUKAEMIA XENOGRAFTS AND PHENOTYPIC EVIDENCE FOR MATURATION

G. PALÚ, P. SELBY, R. POWLES AND P. ALEXANDER*

*From the Institute of Cancer Research, Sutton, Surrey, England*

Received 26 February 1979 Accepted 22 June 1979

**Summary.—A population of human AML cells which have a characteristic karyotypic marker was cryopreserved and then grown in short-term liquid culture for 2 weeks, during which time the cells increased about 7-fold in number and progressively acquired characteristics of macrophages. $10^7$ cells obtained after 1 day in culture, when they were almost devoid of Fc receptors ($Fc^-$), on inoculation into immune-deprived mice gave rise to tumours in more than 90% of the animals. However, after 13 days of culture, when almost all the cells had Fc receptors ($Fc^+$), a similar inoculum did not grow as tumours. After 7 days in culture the cells were heterogeneous, and divided about equally into $Fc^+$ and $Fc^-$ cells, both of which were replicating. The $Fc^-$ population was capable of producing tumours, whereas the $Fc^+$ was not. Of 23 assessable xenograft tumours produced by the AML cells, 14 regressed completely, 4 grew progressively and 5 grew progressively after initial regression. Progressive tumours could be further transplanted. The regressions may arise as a result of maturation *in vivo* similar to that seen *in vitro*.**

A POPULATION of cryopreserved human AML cells was available in our laboratory which grew proliferatively for 2 weeks in liquid culture. The detailed histochemical enzymatic and surface characteristics of these cells and their progressive changes in culture are the subject of a comprehensive report (Palú *et al.*, 1979). We showed that dividing cells in culture, regardless of phenotype, carried a characteristic abnormality of Chromosome Number 11. The purpose of this investigation was to examine whether it was possible to produce transplantable xenografts from this cell population and to compare *in vivo* tumorigenicity with the gradual maturation towards macrophages which occurs *in vitro*. In earlier investigations, Franks *et al.* (1977) had reported that AML cells taken directly from patients gave rise to small tumours in immune-deprived mice, but that these regressed. A further objective of this study was, therefore, to determine whether progressively growing xenografts could be obtained from AML cells grown for short periods *in vitro* and not having the properties of established tissue-culture cell lines.

### MATERIALS AND METHODS

*AML cells.*—One population of AML cells was studied. They were removed using a Blood Cell Separator (Powles *et al.*, 1974) from an untreated 17-year-old female with AML, whose WBC count was $96{\cdot}6 \times 10^9$/l. They were then stored in liquid $N_2$ and karyotyped as described in Chapuis *et al.* (1977) and Palú *et al.* (1979).

Culture conditions were as described in those papers.

*Technique for proliferative cultures of AML cells.*—Cultures were established in 35mm Petri dishes as described by Palú *et al* (1979). The cells growing in suspension were counted with a standard haemacytometer. Cells adherent to the plate were enumerated *in situ* with a calibrated objective, or alternatively

* To whom correspondence should be addressed.

after the nuclei had been detached from the cytoplasm with 0·1M citric acid.

*Cells synthesizing DNA*.—Their number was estimated after incubation with [3H]-TdR at 1 μCi/ml (37 KBq/ml) (sp. act. 20/30 Ci/mM (740–1110 GBq/mM)) for 3 h. Autoradiographs were performed on fixed cells using a conventional dipping technique and Ilford K 5 emulsion. Films were exposed in the dark for 7–10 days, developed and stained with Giemsa. At least 500 cells were counted to determine the labelling index.

*Fc receptors* (*EA rosettes*).—Non-adherent cells forming rosettes with sensitized sheep erythrocytes were determined as previously described by Palú *et al.* (1979).

*Separation of $Fc^+$ from $Fc^-$ cells*.—Some of the cells growing in suspension adhered to the plastic culture dish in absence of serum, and those cells which remained non-adherent under these conditions were wholly $Fc^-$. If, to the cells which had adhered to plastic in the absence of serum, serum is added for ~12 h at 37°C, the majority of the cells detached and more than 90% of these were $Fc^+$. This simple method of separation was preferred to other fractionation procedures to obtain large numbers of cultured AML cells which were $Fc^+$ or $Fc^-$ respectively, to study their DNA synthetic capacity and growth in animals.

*Xenografts*.—Male CBA mice were used, which had been rendered immuno-deprived by neonatal thymectomy, followed after 2–4 weeks by a priming dose of Ara-C 200 mg/kg and 900R total body irradiation as described (Steel *et al.*, 1978; Millar *et al.*, 1978). The animals were prepared by Mr E. M. Merryweather of the Division of Biophysics animal department. AML cells were injected s.c. in the dorsal anterior region of the animals, after having been cultured for 1, 7, 13 days and on occasion separated into $Fc^+$ and $Fc^-$ populations by adherence. Tumour growth was measured with a caliper, recording 2 diameters at right angles of the s.c. nodules.

*Cellular composition of xenograft tumours*.—After removal of growths of between 0·3 and 0·7 cm diameter, they were cut into small fragments and a cell suspension made by dissociation in 0·1% trypsin, 0·1% collagenase and 0·01% DNAse. The cells so obtained were tested for Fc receptors immediately after enzymatic dispersion and after 24h culture. The percentage of mouse cells present within these tumours was assayed using a CBA anti-human serum in complement-dependent lysis. The antiserum was obtained after multiple immunization of normal male CBA with the same AML population used for tumour induction in the immune-deprived CBA. Lyophilized rabbit serum served as the source of complement, after adsorption with AML cells. Cytochemical studies were as described by Palú *et al.* (1979).

RESULTS

### In vitro *maturation and proliferation*

The figure shows that the AML cells recovered from cryopreservation proliferate in liquid culture and increase in number about 7-fold over a period of 2 weeks. The culture consists of 2 types of cells, those that remain in suspension and those

FIG.—Maturation of AML cells in a 3ml liquid culture. ▲ total number of cells. ○ $Fc^+$ non-adherent cells (enumerated by a haemacytometer count. △ $Fc^-$ non-adherent cells. ● cells adhering to the Petri dish and enumerated by counting nuclei detached by citric acid treatment. The initial inoculum was $10^6$ viable cells recovered from cryopreservation.

TABLE I.—*DNA synthesis of AML cells in short-term culture*

| Days in culture | Cell sample | Labelling index (%) |
|---|---|---|
| 1 | Total | 30 |
| 7 | $Fc^+$ and $Fc^-$ (non-adherent) | 20 |
| 7 | $Fc^+$ | 14 |
| 7 | $Fc^-$ | 31 |
| 13 | Total non-adherent | 4 |
| 13 | Adherent | < 0·5 |

which adhere firmly to the culture vessel. Significant numbers of adherent cells only become apparent after 7 days of culture, and constitute 20% of the total cell population by Day 13. The non-adherent population undergoes progressive change during culture as demonstrated by the appearance of Fc receptors. Initially, $Fc^+$ cells constitute a small proportion, but eventually 90% of all the non-adherent cells acquire Fc receptors (all the adherent cells are always $Fc^+$). Table I shows that both $Fc^-$ and $Fc^+$ non-adherent cells synthesize DNA, and inspection of the figure shows that $Fc^+$ non-adherent cells contribute to the increase in cell numbers. The adherent cells have a very low labelling index; in experiments (not reported) in which adherent cells alone were cultured, there was no DNA synthesis or increase in cell number. We conclude that the pattern of maturation is first the acquisition of Fc receptors, and these cells retain the capacity to divide *in vitro* and retain the characteristic karyotypic abnormality. Subsequently they acquire macrophage-like properties as described first by Balkwill and Oliver (1976).

*Xenografts*

Table II shows that the AML cells after 24 h culture, when only 10% carried Fc receptors, (90% $Fc^-$ cells) consistently produced nodules when inoculated into immuno-deprived mice. When the number of inoculating cells was less than $10^7$, the cells grew as xenografts in 64% of the animals (7/11). However, if $10^7$ or more cells were inoculated tumours were almost invariably obtained (92%; = 23/25).

Whilst most of the tumours regressed spontaneously, we observed (unlike Franks, 1977) that after inoculation of $10^7$ cells 40% of the assessable tumour nodules grew progressively, although half of these showed an initial regression (see Table II). Progressively growing tumours could be further transplanted. Morphologically these tumours were human AML cells, with many mitotic figures showing the characteristic chromosome marker of this population. The nodules were dispersed enzymatically, and more than 95% of the resulting cells were lysed by a mouse anti-human serum in the presence of complement.

TABLE II.—*Growth of AML cells after different periods of culture in immune-deprived mice*

| Length of culture (days) | Cell sample | Inoculum (cell no.) | No of mice inoculated | Takes* | Regressors† | Progressors‡ |
|---|---|---|---|---|---|---|
| 1 | | $1 \times 10^6$ | 3 | 2 | 1/2 | 1 |
| | | $3 \times 10^6$ | 4 | 2 | 2/2 | 0 |
| | | $7 \times 10^6$ | 4 | 3 | 3/3 | 0 |
| | | $1 \times 10^7$ | 25 | 23 | 14/23 | 9 |
| 7 | Total (~50% $Fc^+$) | $1 \times 10^7$ | 7 | 3 | 3/3 | 0 |
| | $Fc^-$ | $1 \times 10^7$ | 7 | 6 | 6/6 | 0 |
| | $Fc^+$ | $1 \times 10^7$ | 4 | 0 | 0 | 0 |
| 13 | ~90% $Fc^+$ | $1 \times 10^7$ | 10 | 0 | 0 | 0 |
| 1<br>13 | | $1 \times 10^7$<br>$5 \times 10^6$ } | 3 | 3 | 0 | 3 |

* A nodule exceeding 5 mm in diameter appearing within 3 weeks of inoculation.
† The total disappearance of a nodule which occurred within 2–5 weeks of inoculation.
‡ Continuous growth for more than 60 days, leading to a large lesion which required that the mouse be killed.

TABLE III.—*Properties of cells derived from AML xenografts which developed from an inoculum of* $10^7$ *AML cells cultured for* 1 *day*

| Time of excision after implantation (weeks) | No. of animals | % Mouse | % $Fc^+$ | % non-specific esterase$^+$ |
|---|---|---|---|---|
| 3* | 3 | 1 | 70 | 40 |
| 17† | 2 | 1 | 9 | 15 |
| Inoculated cells | | | 9 | 20 |

* When nodules were regressing.

† Progressively growing tumours.

Cells cultured for 7 days grew as xenografts in 3/7 mice (Table II). The inoculated cells consisted of about equal numbers of $Fc^+$ and $Fc^-$ cells, both of which, as shown in Table I, synthesized DNA. After separation by *in vitro* adherence into 2 populations which were respectively $>90\%$ $Fc^+$ and $<10\%$ $Fc^+$, only the $Fc^-$ cells grew as xenografts. In each of the 6 instances, the tumours derived from the cells that had been cultured for 7 days regressed even though the inoculum consisted of $10^7$ cells.

After 13 days of culture the non-adherent cells which were viable (*i.e.* excluded trypan blue) and looked morphologically normal failed to grow as xenografts in 10 animals tested. More than 90% of these cells were $Fc^+$ and 70% were positive for nonspecific esterase activity. However, these $Fc^+$ cells did not appear to inhibit the growth *in vivo* of the $Fc^-$ cells, since in one experiment involving 3 animals, $10^7$ $Fc^-$ (Day 1 culture) cells when inoculated admixed with $5 \times 10^6$ $Fc^+$ (Day 13) cells grew as xenografts (see Table II).

Table III provides some evidence that the regression of xenografts is accompanied by maturation. Cells derived from tumours which had passed their maximum size of $\sim 7$ mm in diameter, and at excision were in the range of 4 cm in diameter, had a greatly increased incidence of $Fc^+$ and nonspecific-esterase-positive cells than the original inoculum. Maturation was much less evident in the 2 progressively growing tumours studied.

### DISCUSSION

Franks *et al.* (1977) found that AML cells taken directly from the blood of patients, grew in thymectomized, irradiated mice reconstituted with marrow as s.c. tumours for 2–3 weeks, and then regressed. We find that a population of cryopreserved AML cells will take as xenografts in 90% of mice that had been immune-deprived by a procedure that did not involve marrow grafting, and a chromosome marker shows that the dividing cells in the tumour were of leukaemic origin. Using a suitable anti-human mouse serum it was found that there are very few mouse cells in these tumours. 40% of the nodules grow progressively and can be transplanted into other immune-deprived animals, in which they grow progressively. Maturation of the AML cells in culture, as revealed by acquisition of Fc receptors, is associated with loss of tumorigenicity. Thus $Fc^+$ cells obtained from proliferative cultures after 9–14 days in liquid media fail to grow as xenografts in spite of the fact that the cells divide (Table I).

A remarkable aspect of the growth as xenografts of the AML cells, either when taken directly from the patient or from short-term culture, is the high incidence of spontaneous regression of the nodules after 2–3 weeks. The regression of these $Fc^-$ AML cells is unlikely to be caused by an immune rejection, since if this were the case we would anticipate an inverse relationship between the size of the cell inoculum and the incidence of regression, but this is not found (see Table II). But, more importantly, mice that had been inoculated and developed a tumour which regressed, when rechallenged with $10^7$ $Fc^-$ cells developed tumours which then again regressed in 2 of the 3 mice in which this experiment was performed. This pattern is not compatible with an interpretation of the regression as an immune rejection caused by the partial recovery of immune function of the mice with time.

Some evidence that the regression of the xenografts is associated with maturation similar to that occurring *in vitro* is provided by experiments measuring Fc receptors of the cells in the xenografts before rejection. The percentage of $Fc^+$ cells determined immediately and after 24h of culture showed that up to 70% of the cells were $Fc^+$, although less than 10% were $Fc^+$ when inoculated. The cells derived from the xenograft tumours also appeared more mature than the inoculated cells, as they had an increased nonspecific esterase staining, a change which is also seen after 7 days of *in vitro* culture. The experiments reported show that this population of AML cells, studied and identified karyotypically, undergoes *in vitro* progressive changes towards a macrophage phenotype which is unable to divide. At an intermediate stage in the *in vitro* cultures (7 days) some cells have acquired Fc receptors, and these do not then grow as tumours in immune-deprived mice, in spite of retaining the capacity to divide *in vitro*. The spontaneous regression of the AML xenografts may also be the result of this differentiation. These findings raise the possibility that interference with maturation may be a component of AML in man.

This investigation has been supported by grants from the Leukaemia Research Fund. We wish to thank Dr Sylvia Lawler for assistance with the karyotyping and Dr Gordon Steel for making the immune-deprived mice available to us.

### REFERENCES

BALKWILL, F. R. & OLIVER, R. T. D. (1976) Diagnostic and prognostic significance of peripheral blood cultural characteristics in adult acute leukaemia. *Br. J. Cancer*, **33**, 400.

CHAPUIS, B., SUMMERSGILL, B. M., COCKS, P. & 4 others (1977) Test for cryopreservation efficiency of human acute myelogenous leukaemia cells relevant to clinical requirements. *Cryobiology*, **14**, 637.

FRANKS, C. R., BISHOP, D., BALKWILL, F. R., OLIVER, R. T. D. & SPECTOR, W. G. (1977) Growth of acute myeloid leukaemia as discrete subcutaneous tumours in immune-deprived mice. *Br. J. Cancer*, **35**, 697.

MILLAR, J. L., BLACKETT, N. M. & HUDSPITH, B. N. (1978) Enhanced post-irradiation recovery of the haemopoietic system in animals pre-treated with a variety of cytotoxic agents. *Cell Tissue Kinet.*, **11**, 543.

PALÚ, G., POWLES, R., SELBY, P., SUMMERSGILL, B. M. & ALEXANDER, P. (1979) Patterns of maturation in short-term culture of human acute myeloid leukaemia cells. *Brit. J. Cancer*, **40**, 720.

POWLES, R. L., LISTER, T. A., OLIVER, R. T. D. & 5 others (1974) Safe method of collecting leukaemia cells from patients with acute myelogenous leukaemia for use as immuno-therapy. *Br. Med. J.*, iv. 375.

STEEL, G. G., COURTENAY, U. D. & ROSTOM, A. Y. (1978) Improved immune-suppression techniques for the xenografting of human tumours. *Br. J. Cancer*, **37**, 224.